# Regularized survival learning and cross-database analysis enabled identification of colorectal cancer prognosis-related immune genes

**DOI:** 10.3389/fgene.2023.1148470

**Published:** 2023-02-23

**Authors:** Dongmei Ai, Mingmei Wang, Qingchuan Zhang, Longwei Cheng, Yishu Wang, Xiuqin Liu, Li C. Xia

**Affiliations:** ^1^ School of Mathematics and Physics, University of Science and Technology Beijing, Beijing, China; ^2^ National Engineering Laboratory for Agri-Product Quality Traceability, Beijing Technology and Business University, Beijing, China; ^3^ School of Mathematics, South China University of Technology, Guangzhou, China

**Keywords:** LASSO, multivariate cox analysis, prognosis, immune gene, colorectal cancer

## Abstract

Colon adenocarcinoma is the most common type of colorectal cancer. The prognosis of advanced colorectal cancer patients who received treatment is still very poor. Therefore, identifying new biomarkers for prognosis prediction has important significance for improving treatment strategies. However, the power of biomarker analyses was limited by the used sample size of individual database. In this study, we combined Genotype-Tissue Expression (GTEx) and The Cancer Genome Atlas (TCGA) databases to expand the number of healthy tissue samples. We screened differentially expressed genes between the GTEx healthy samples and TCGA tumor samples. Subsequently, we applied least absolute shrinkage and selection operator (LASSO) regression and multivariate Cox analysis to identify nine prognosis-related immune genes: *ANGPTL4*, *IDO1*, *NOX1*, *CXCL3*, *LTB4R*, *IL1RL2*, *CD72*, *NOS2*, and *NUDT6*. We computed the risk scores of samples based on the expression levels of these genes and divided patients into high- and low-risk groups according to this risk score. Survival analysis results showed a significant difference in survival rate between the two risk groups. The high-risk group had a significantly lower overall survival rate and poorer prognosis. We found the receiver operating characteristic based on the risk score was showed to accurately predict patients’ prognosis. These prognosis-related immune genes may be potential biomarkers for colorectal cancer diagnosis and treatment. Our open-source code is freely available from GitHub at https://github.com/gutmicrobes/Prognosis-model.git.

## 1 Introduction

According to global cancer statistics 2020 data, colorectal cancer ranked third by cancer incidence and second by cancer mortality rate (Sung et al., 2021). According to predictions, the number of new colorectal cancers will reach 2.2 million and deaths will reach 1.1 million in 2030 (Arnold et al., 2017). Colorectal cancer usually occurs in the inner walls of the colon or rectum (Lao and Grady, 2011). When malignant cells are formed in the colon or rectum, it will lead to the occurrence of colorectal cancer (Wang et al., 2021). Based on histological classification, colon adenocarcinoma is the main type of colorectal cancer (Wei et al., 2018). The main causes of transformation of normal colonic epithelium to colon adenocarcinoma are genetic and epigenetic changes (Coppede, 2014). At present, the main method for treating colon adenocarcinoma is surgery combined with postoperative chemotherapy (Hashiguchi et al., 2020; Tarazona et al., 2020). Even with standard treatment, the outcomes of advanced colon adenocarcinoma patients are still very poor and varies widely (Andre et al., 2004; Nishihara et al., 2013; Sadanandam et al., 2013). Therefore, using simple conventional factors, such as clinicopathology stage, is insufficient for accurate prognostic prediction of colon adenocarcinoma patients, which calls for the discovery of new biomarkers to predict the prognosis of patients and improve treatment outcomes.

Biomarkers improve patients’ prognosis by treating patients who may benefit from a given treatment (Blangero et al., 2020). In recent years, the rapid development of bioinformatics tools has enabled researchers to rapidly identify colorectal cancer biomarkers based on differentially expressed genes (DEGs). For examples, Dalerba et al. found that *CDX2* is a prognostic biomarker and that *CDX2* deletion is associated with poor prognosis in stage II or III colorectal cancer patients (Dalerba et al., 2016). Li et al. found that the immune gene *ULBP2* is a prognostic biomarker and that *TMEM37* and *GRP* may also be potential prognostic genes for colon cancer (Li et al., 2018). Wang et al. found that *MXRA5* is aberrantly expressed in colorectal cancer tissues and is a biomarker for the early detection of colorectal cancer (Wang et al., 2013). Den Uil et al. found that *KCNQ1* is a prognostic biomarker for predicting recurrence in stage II and III colon cancer patients (den Uil et al., 2016). Woischke et al. found that *CYB5R1* is intimately associated with poor prognosis in colorectal cancer (Woischke et al., 2016). Kandimalla et al. found that methylated *AXIN2* and *DKK1* are useful biomarkers for recurrence in stage II colon cancer patients (Kandimalla et al., 2017).

Compared with a single biomarker, combining multiple biomarkers in a model can predict patients’ prognosis more accurately (Qu et al., 2018). For example, Lin et al. proposed a new prognosis risk score characteristic based on nine long non-coding RNAs (lncRNAs) associated with colon cancer prognosis (Lin et al., 2020). This characteristic has important clinical significance in improving the prediction results of colon cancer patients, and these lncRNAs as a whole may be biomarkers that affect prognosis. Zuo et al. carried out univariate and multivariate Cox analysis to identify six DEGs associated with colorectal cancer patients prognosis, including *EPHA6*, *TIMPI*, *IRX6*, *ART5*, *HIST3H2BB*, and *FOXD1* (Zuo et al., 2019). Their combined is an independent biomarker for predicting the survival rate.

Currently, immunotherapy has demonstrated huge potential in improving tumor prognosis, and studies have increasingly shown that expression of immune-related genes may be related to cancer patients’ prognosis (Galon et al., 2013; Bedognetti et al., 2015). For example, Miao et al. identified 12 immune genes (*SLC10A2*, *CXCL3*, *NOX4*, *FABP4*, *ADIPOQ*, *IGKV1-33*, *IGLV6-57*, *INHBA*, *UCN*, *VIP*, *NGFR*, and *TRDC*) associated with the prognosis of colon adenocarcinoma patients (Miao et al., 2020). The associated risk score proved an independent prognostic factor. Therefore, the identification of colon adenocarcinoma-related immune genes is particularly useful to promote the development of tools to carry out colon adenocarcinoma immunotherapy.

However, the aforementioned studies only used healthy samples and tumor samples from The Cancer Genome Atlas (TCGA) database to identify DEGs between healthy samples and tumor samples. The differences in the number of samples in the TCGA database are very large. For example, several hundred tumor samples are available, but only a few dozen healthy samples (Mounir et al., 2019). This big difference will lead to inaccuracy in the identification of DEGs.

Therefore, in this study, we collected healthy tissue samples from the Genotype-Tissue Expression (GTEx) database and tumor tissue samples from the TCGA database when screening for DEGs. Large sample size enabled us to sensitively identify biomarkers based on DEGs. We employed least absolute shrinkage and selection operator (LASSO) regression and multivariate Cox analysis to construct a risk model based on multiple immune genes. This model can accurately predict patients’ prognosis (AUC of training dataset >0.8), which has important clinical significance. The immune genes identified in the model could be used as potential biomarkers.

## 2 Materials and methods

### 2.1 Data sources

Healthy colon tissue RNA-seq data of 308 samples in the GTEx database were downloaded from the UCSC website (https://xenabrowser.net/, accessed on 25 March 2022), as fragments per kilobase of exon model per million mapped fragments (FPKM) values. Gene expression data were extracted from 308 healthy samples. We removed low-expressing genes that the mean expression level is less than 0.2. After removing low-expressing genes, the expression levels of 22,116 genes were retained.

**FIGURE 1:**
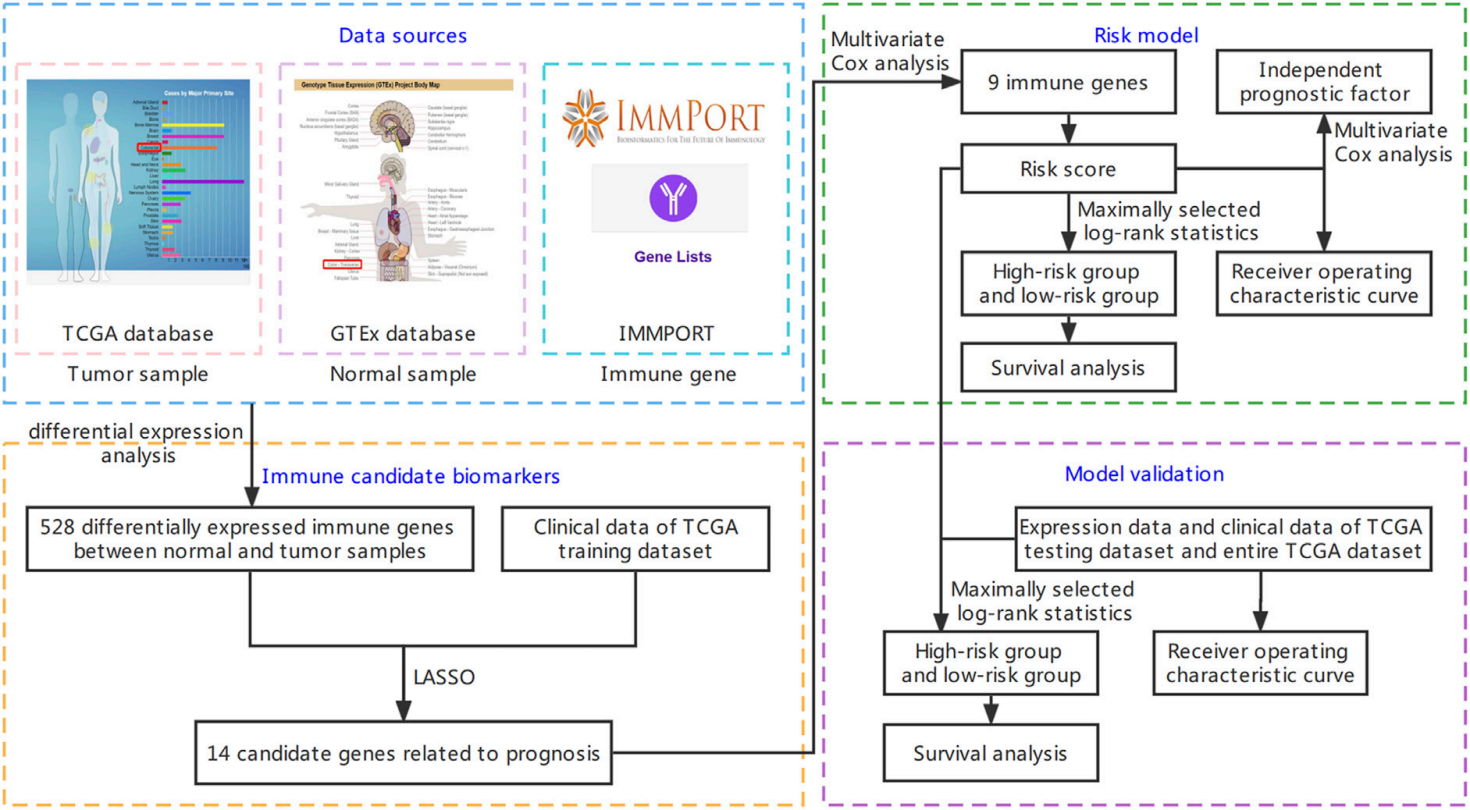
Flow chart of this study. It is mainly divided into four parts: downloading data, screening immune candidate biomarkers, building risk model, and model validation. The detailed steps are shown in the figure.

The RNA-seq FPKM data of 391 colon adenocarcinoma samples were downloaded from the TCGA website (https://portal.gdc.cancer.gov/, accessed on 21 March 2022). Genes (mean expression level <0.2 in samples) were removed to obtain the expression levels of 14,791 genes. The clinical data of 391 colon adenocarcinoma patients were also downloaded from the TCGA website. The analysis flow chart is shown in Figure 1.

### 2.2 Screening of differentially expressed genes

The list of human immune genes was downloaded from the Immunology Database and Analysis Portal (IMMPORT) database (https://www.immport.org/home, accessed on 30 March 2022). Total 1793 immune genes were included. The GTEx dataset and TCGA dataset were combined to obtain 14,306 intersection genes. We used R package “limma” to screen DEGs between healthy samples and tumor samples through Wilcoxon test (Ritchie et al., 2015). False discovery rate (fdr) was computed to correct multiple testing. The screening criteria were fdr<0.05 and $\left|\log_{2}\left(fold\;change\right)\right|>1$ . After obtaining the list of DEGs, the intersection with immune genes was obtained as differentially expressed immune genes.

$$\log_{2}\left(fold\;change\right)=\log_{2}\frac{mean\;value\;of\;gene\;in\;tumor\;group}{mean\;value\;of\;gene\;in\;healthy\;group}$$ (1)

### 2.3 Regularized survival analysis

Univariate Cox analysis is typically used to screen for prognosis-related genes in patients, and then a multivariate model is constructed to further confirm whether the association between gene and survival is independent. However, this method does not consider the multiple collinear effects between genes, and contradiction in hazard ratios (HR) obtained from univariate Cox regression and multivariate Cox regression occurs, causing model distortion. However, the multivariate analysis also suffers from the curse of dimensionality when the number of genes is greater than the sample size.

The modernized regularized survival analysis approach, such as LASSO, avoids the high-dimensionality issue by soft-selecting significant features. We thus employed LASSO Cox regression for gene screening before multivariate Cox regression model was used to establish prognostic characteristics. LASSO regularization, which was proposed by Tibshirani (Tibshirani, 1997), uses L1 norm for the shrinkage penalty in which the coefficients of not-so-important genes are compressed to 0, while the coefficients of important genes are retained at more than 0. This decreases the number of covariates in the Cox regression (i.e., genes). Genes with a coefficient >0 in LASSO-Cox regression were selected for further calculation of the risk score (Kidd et al., 2018). The formula of LASSO is as follows (Emmert-Streib and Dehmer, 2019):

$$\hat{\beta }=\arg\min\left\{\frac{1}{2n}\overset{n}{\underset{i=1}{\sum }}{\left(y_{i}-\underset{j}{\sum }\beta_{j}x_{ij}\right)}^{2}+\lambda {\left\|\beta \right\|}_{1}\right\}=\arg\min\left\{\frac{1}{2n}{\left\|y-X\beta \right\|}_{2}^{2}+\lambda {\left\|\beta \right\|}_{1}\right\}$$ (2)

The survival data of TCGA patients and the expression data of differentially expressed immune genes were combined. The 391 patient samples were randomized into a training dataset and a validation dataset. The training dataset accounted for 70% (273 samples) of the dataset, and the testing dataset accounted for 30% (118 samples) of the dataset. Data in the training dataset were used for LASSO regression. We used R package “glmnet” to conduct LASSO regression analysis. The objective was to minimize overfitting, i.e., removal of genes that will cause overfitting, and select differentially expressed immune genes significantly associated with survival.

### 2.4 Multivariate Cox analysis

The multivariate Cox regression model, also known as the proportional hazards model, is a semi-parametric regression model (Kleinbaum and Klein, 2012). In this model, survival outcome and survival time were used as dependent variables. The model can simultaneously analyze the effects of multiple variables (e.g., genes) on survival. Candidate immune genes related to prognosis were obtained through LASSO analysis, and then a risk model was constructed through multivariate Cox analysis. Multivariate Cox analysis will screen candidate immune genes by stepwise regression method. Multivariate Cox analysis was conducted using the R package “survival”.

A multivariate Cox regression model was used to construct a prognostic characteristic of immune genes and calculate the risk score of each patient sample. The calculation formula is as follows:

$$Risk\;score=\overset{n}{\underset{i=1}{\sum }}\exp_{i}*{coef}_{i}$$ (3)

where n is the number of characteristic genes included in the model, $\exp_{i}$ represents the expression level of gene i , and ${coef}_{i}$ represents the coefficient of gene i in the multivariate Cox regression analysis. We determined the optimal cut-off value of risk score according to the maximally selected log-rank statistics (Wright et al., 2017). Patients were divided into two groups based on the optimal cut-off value. Patients with risk scores greater than the cut-off value were included in the high-risk group, and patients whose risk scores did not exceed the cut-off value were included in the low-risk group.

### 2.5 Survival analysis and ROC curve computing

The Kaplan-Meier curve is also known as the survival curve and is a commonly used method in survival analysis. The Kaplan-Meier curve mainly analyzes the effect of a single factor on survival, and it is used to estimate the survival rate of patients. Survival time is the *x*-axis, survival rate is the *y*-axis, and a continuous stepped curve is computed to describe the relationship between survival time and survival rate. The log-rank test was used to evaluate survival differences between the two groups. We used the R package “survival” to conduct survival analysis. Receiver operating characteristic (ROC) curves were computed, and the area under the ROC curve (AUC) was calculated to assess the accuracy of the prognostic model. We used the R package “time ROC” package to calculate the AUC at different cutoff times.

### 2.6 Independence and model validation

Multivariate analysis was carried out for patient samples with clinical characteristics, and the prognostic value of the risk score was assessed. Based on multivariate analysis, the characteristics of p<0.05 can be used as an independent prognostic factor. The entire TCGA dataset (391 samples) and testing dataset (118 samples) were used for model validation. The risk score of each sample was calculated based on the same formula [see Formula (4)], and samples were grouped into high- and low-risk groups based on the optimal cut-off value. Survival analysis was performed for these two groups to evaluate the survival differences between the two groups. A ROC curve was computed, and the AUC was calculated to assess model accuracy. Data analysis and visualization were performed using R software (version 4.1.3, https://www.rstudio.com/, accessed on 18 March 2022).

## 3 Results

### 3.1 Screening candidate immune biomarker

The Wilcoxon test was used to screen DEGs between GTEx healthy samples and TCGA tumor samples, and the screening criteria were fdr<0.05 and $\left|\log_{2}\left(fold\;change\right)\right|>1$ . By comparing with the healthy tissue group, 7670 DEGs were obtained. Among these, 6381 genes were downregulated, and 1289 genes were upregulated. A listing of 1793 immune genes was downloaded from the IMMPORT database, and the intersection with DEGs, which contained 528 differentially expressed immune genes, was retained. Among these, 383 genes were downregulated, and 145 genes were upregulated.

Clinical data of 391 colon adenocarcinoma patients were downloaded from the TCGA database. The clinical information of 341 samples was retained by deleting some samples with unknown clinical characteristics. Table 1 shows the detailed clinical information. We divide the sample into two groups according to age, one group is no more than 60 years old, and the other group is over 60 years old (Lin et al., 2019).

**TABLE 1:** Summary of the clinical data of The Cancer Genome Atlas (TCGA) colon adenocarcinoma patients.

Clinical parameter	Variable	n (total = 341)	Percentage (%)
Age (years)	≤60	97	28.4
	>60	244	71.6
Gender	Female	155	45.5
	Male	186	54.5
Stage	Stage Ⅰ	59	17.3
	Stage Ⅱ	138	40.4
	Stage Ⅲ	93	27.3
	Stage Ⅳ	51	15.0
Tumor	T1	8	2.4
	T2	57	16.7
	T3	236	69.2
	T4	40	11.7
Metastasis	M0	290	85.0
	M1	51	15.0
Lymph Node	N0	203	59.5
	N1	81	23.8
	N2	57	16.7
Survival status	Alive	282	82.7
	Dead	59	17.3

TNM staging system is the most commonly used tumor staging system in the world. T is the first letter of “Tumor”, referring to the tumor size and local invasion range. T1 refers to the smaller primary part. T2 refers to the larger primary part. T3 refers to the larger primary part and/or the infiltration exceeds the edge of the primary organ. T4 refers to the very large primary part and/or the infiltration to adjacent organs. N is the first letter of “Node” in the lynch node, which refers to the involvement of regional lymph nodes. N0 refers to no lymph node metastasis. N1 refers to local lymph node metastasis. N2 refers to extensive lymph node metastasis. M is the first letter of “metastasis”, which refers to remote metastasis. M0 means no distal metastasis, and the tumor does not spread to other parts of the body. M1 refers to distal metastasis, and the tumor spreads to other parts of the body. Stage group determined from clinical information on the tumor (T), regional node (N) and metastases (M) and by grouping cases with similar prognosis for cancer. Stage includes stage Ⅰ, stage Ⅱ, stage Ⅲ and stage Ⅳ. Stage Ⅰ tumors are usually relatively early tumors with relatively good prognosis. The higher the stage, the higher the degree of tumor progression.

Expression and survival data of differentially expressed immune genes were combined to obtain the expression and survival data of differentially expressed immune genes of 391 samples. The 391 samples were randomized into the training dataset and testing dataset. The sample size of the training dataset accounted for 70% (273 samples) of the total sample size, and the sample size of the testing dataset accounted for 30% (118 samples) of the total sample size. To determine prognosis-related immune genes, training dataset samples were used for LASSO regression. Among the 528 differentially expressed immune genes between the healthy and tumor samples, 14 candidate genes were obtained (Supplementary Figure S1).

### 3.2 Predictive model construction through Multivariate Cox analysis

Multivariate Cox analysis was used for further screening of the 14 candidate biomarker genes, and nine biomarker genes were finally obtained (Table 2). The expression levels of these nine immune genes and their corresponding correlation coefficients were used to calculate risk scores. The calculation formula is as follows:

$$\begin{array}{c} Risk\;score=\left(0.109*expression\;level\;of\;ANGPTL4\right)\\ +\left(0.005*expression\;level\;of\;IDO1\right)\\ -\left(0.006*expression\;level\;of\;NOX1\right)\\ -\left(0.016*expression\;level\;of\;CXCL3\right)\\ +\left(0.076*expression\;level\;of\;LTB4R\right)\\ +\left(0.133*expression\;level\;of\;IL1RL2\right)\\ +\left(0.304*expression\;level\;of\;CD72\right)\\ -\left(0.018*expression\;level\;of\;NOS2\right)\\ -\left(1.689*expression\;level\;of\;NUDT6\right) \end{array}$$ (4)

**TABLE 2:** Multivariate Cox analysis results of training dataset.

Gene symbol	Coef	Hazard ratios (HR)	95% CI of HR
ANGPTL4	0.109	1.115	1.069–1.163
IDO1	0.005	1.005	1.001–1.009
NOX1	−0.006	0.994	0.988–1.000
CXCL3	−0.016	0.984	0.962–1.007
LTB4R	0.076	1.078	1.010–1.152
IL1RL2	0.133	1.142	0.964–1.354
CD72	0.304	1.355	1.037–1.771
NOS2	−0.018	0.982	0.960–1.005
NUDT6	−1.689	0.185	0.031–1.082

**FIGURE 2:**
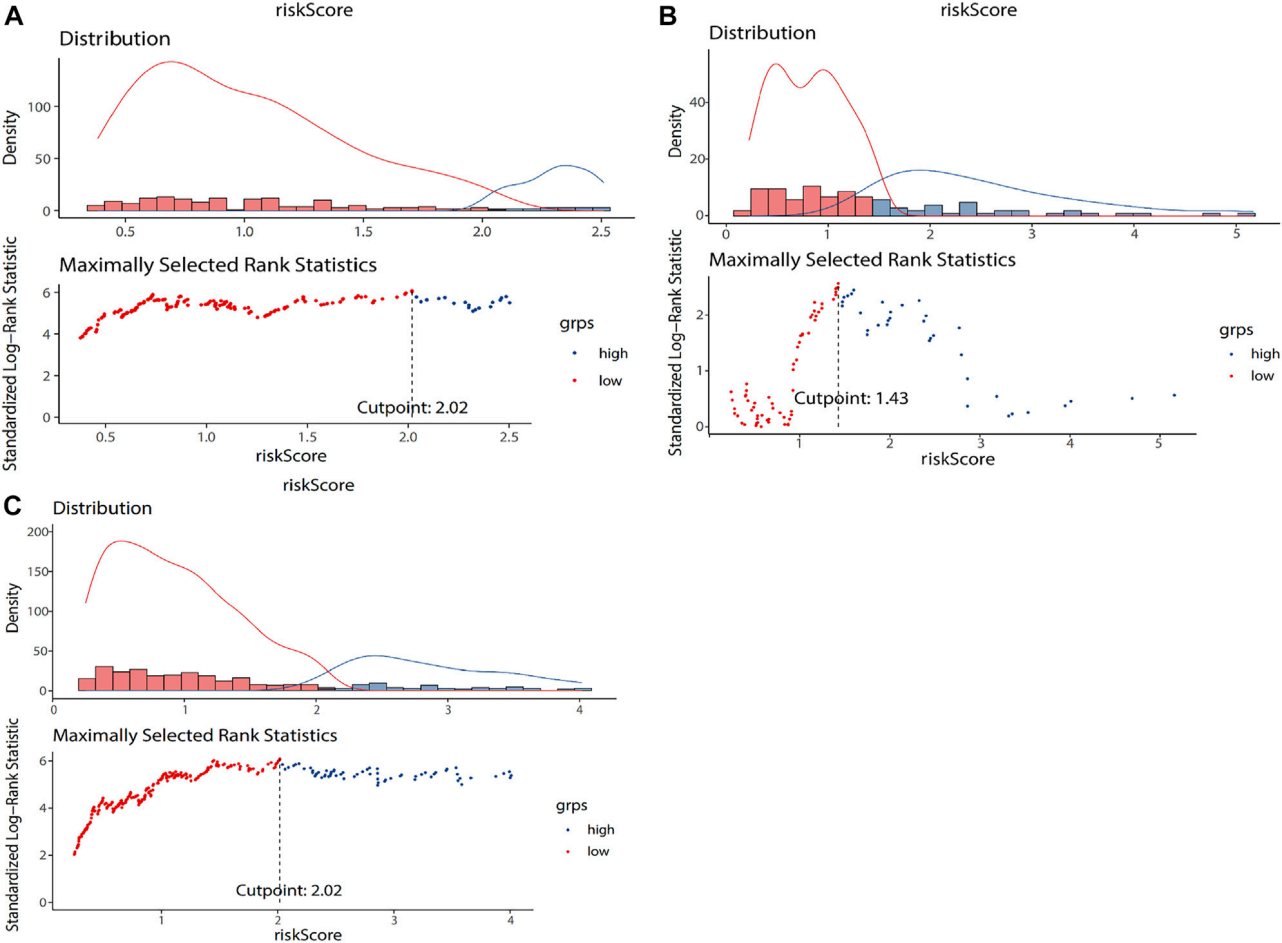
The grouping results of training dataset **(A)**, testing dataset **(B)**, and entire TCGA dataset **(C)**. The lower part of the figure is the optimal cut-off value calculated according to the maximum selection rank statistics. The risk score of the blue dot is lower than the cut-off value, which is a low-risk group. The risk score of the red dot is higher than the cut-off value, which is a high-risk group. The upper part of the figure is the data distribution histogram and density distribution curve of risk score. Blue represents low risk group, and red represents high risk group.

The overall importance of the model was tested. The *p* values of the three tests were less than 0.05, which were likelihood ratio test ( p=1e−10 ), wald test ( p=1e−10 ) and score log rank test ( p<2e−16 ). The optimal cut-off value of risk score is determined through the surv_cutpoint function of R. The optimal cut-off value of training dataset is 2.02 (Figure 2A). The 273 colon adenocarcinoma patients in the training dataset were divided into two groups based on the optimal cut-off value. Patients with risk scores greater than the cut-off were included in the high-risk group (*n* = 73), and patients with risk scores lower than the cut-off were included in the low-risk group (*n* = 200). Supplementary Figure S2 shows the survival distribution of the low- and high-risk groups. As risk score increased, the number of patient deaths increased, and the survival time decreased; that is, the number of deaths in the high-risk group was higher, and the survival rate was lower.

Supplementary Figure S3 shows the heatmap of nine immune genes included in the model. The $\log_{2}\left(expression\;value\right)$ of genes in the healthy and tumor groups are also shown. *ANGPTL4*, *LTB4R*, *CD72*, and *NUDT6* were downregulated, as their expression levels were higher in the healthy group and lower in the tumor group. *IDO1*, *NOX1*, *CXCL3*, *IL1RL2* and *NOS2* were upregulated, as their expression levels were lower in the healthy group and higher in the tumor group.

### 3.3 Survival analysis and ROC characterization of training dataset

**FIGURE 3:**
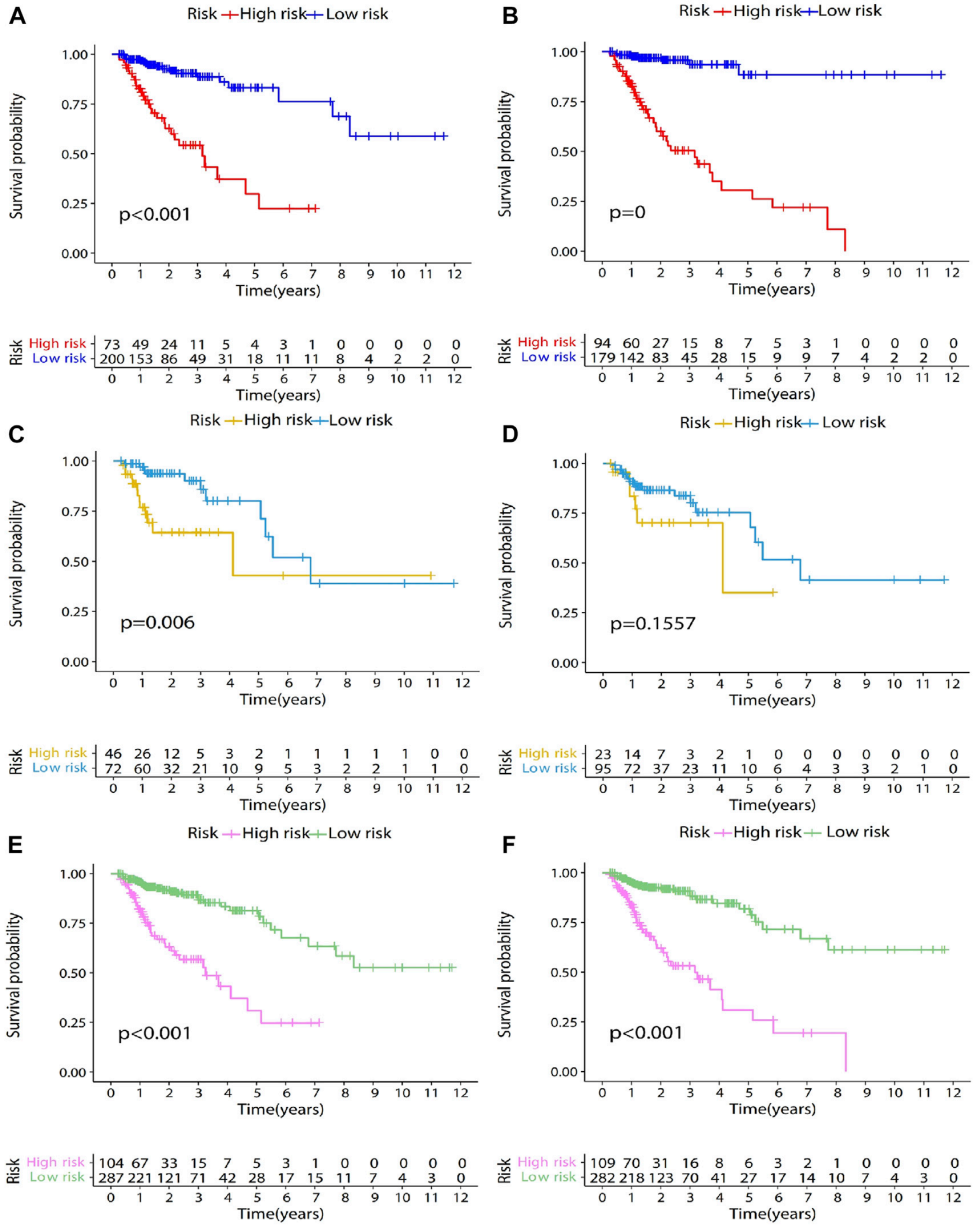
Survival analysis results of training dataset, testing dataset, and entire TCGA dataset. The genes were screened by LASSO regression, and the model was constructed by multifactor cox regression. The survival analysis results of the training dataset, the testing dataset, and the entire TCGA dataset are shown in Figures 3 **(A,C,E)**. After screening the genes through univariate Cox analysis, the survival analysis results of the training dataset, testing dataset and the entire TCGA dataset are shown in Figures 3 **(B,D,F)**. In the survival analysis chart, the x-coordinate represents the survival time, in years. The y-coordinate represents the survival probability. The patients were divided into two groups according to the optimal cut-off value. They are low-risk group and high-risk group. *p*-value represents the difference in survival between the two groups. At the bottom of the figure is a table. The abscissa is the survival time in years. The ordinate is the high-risk group and low-risk group, and the value represents the number of patients remaining at each time point.

The genes were screened by LASSO regression, and the model was constructed by multifactor cox regression. The survival analysis results of the training set, the test set, and the entire data set are shown in Figures 3A,C,E. After screening the genes through univariate Cox analysis, the survival analysis results of the training set, test set and the entire data set are shown in Figures 3B,D,F. Comparing Figures 3C,D, we can see that the survival rate of high-risk group and low-risk group is significantly different without Univariate Cox analysis. Therefore, we choose not to add single factor cox analysis when building the model.

**FIGURE 4:**
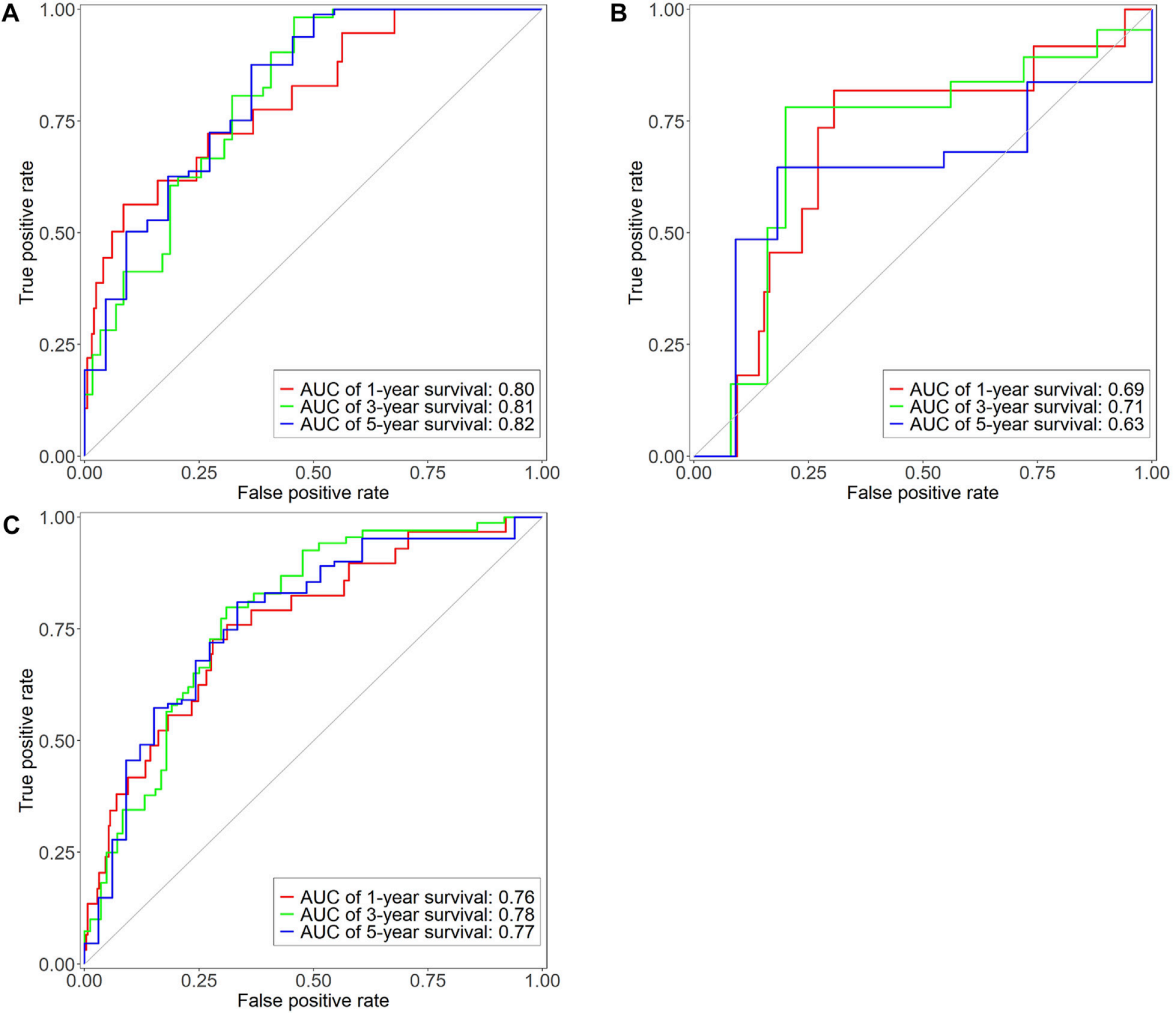
Time-dependent ROC curve of training dataset **(A)**, testing dataset **(B)**, and entire TCGA dataset **(C)**. *X*-axis represents false positive rate, *y*-axis represents true positive rate. Red, green and blue represent the curves of 1 year, 3 years and 5 years respectively.

After patients were divided into high- and low-risk groups, Kaplan-Meier survival analysis was used to compare the survival differences between the two groups. Survival analysis results showed statistically significant difference in survival rate between the high- and low-risk groups ( p<0.001 ; Figure 3A). The high-risk group had lower overall survival rate and poorer prognosis. The median survival was more than 10 years and around 3 years in the low- and high-risk groups, respectively. The three- and 5-year survival rates of the low-risk group were 88% and 80%, respectively. The three- and 5-year survival rates of the high-risk group were 50% and 25%, respectively. The ROC curve was computed to assess the accuracy of the prognostic model. The AUC values of the 1-, 3-, and 5-year overall survival rates were 0.80, 0.81, and 0.82, respectively (Figure 4A), showing that the prognostic model had good accuracy.

### 3.4 Independent prognostic analysis of training dataset

Multivariate analysis was used to evaluate the independent prediction capacity of the model and the clinical characteristics. Clinical data of colon adenocarcinoma patients were downloaded from the TCGA database. Samples with missing clinical data were deleted to obtain 341 samples and their corresponding clinical data, including age, gender, stage, T, M, N, and risk score. Age is used as a numerical variable. Female in gender is represented by 0 and male by 1. Each stage in the T, M, N and stage is represented by corresponding Arabic numerals. Multivariate analysis showed that the *p*-values of age, T, and risk score were all less than 0.05 and were independent prognostic factors (Table 3) that predicted patients’ prognosis.

**TABLE 3:** Multivariate independent prognosis analysis results of training dataset.

Variable	HR	95% CI of HR	*p*-value
Age	1.043	1.012–1.074	∗∗
Gender (Female vs. Male)	0.795	0.415–1.523	ns
Stage	1.062	0.370–3.047	ns
T	2.626	1.274–5.414	∗∗
M	2.155	0.538–8.632	ns
N	1.409	0.717–2.769	ns
Risk score	1.004	1.001–1.007	∗∗

∗∗*p* < 0.01; ns, no significance.

### 3.5 Predictive model validation

The testing dataset (118 samples) and the entire TCGA dataset (391 samples) were used as validation sets for the prognostic model to evaluate model accuracy. The testing dataset included 118 colon adenocarcinoma patient samples. The risk score of each sample was calculated based on the same formula (Formula (4). The optimal cut-off value of risk score is determined through the surv_cutpoint function of R. The optimal cut-off value of testing dataset is 1.43 (Figure 2B). The optimal cut-off value was used to divide 118 patient samples into two groups, namely, the high- (*n* = 46) and low-risk groups (*n* = 72). Kaplan-Meier survival analysis was used to compare survival differences between the two groups. Survival analysis results showed differences in survival rate between the two groups ( p<0.05 ; Figure 3C). Overall survival of the high-risk group was lower, and the prognosis was worse. Median survival was more than 6 and 4 years in the low- and high-risk groups, respectively. The three- and 5-year survival rates of the low-risk group were 86% and 70%, respectively, while the three- and 5-year survival rates of the high-risk group were <65% and <40%, respectively. The reason for the intersection of survival curves at the end may have resulted from the low sample size. Figure 4B shows the ROC curve of the testing dataset. The AUC of the 3-year overall survival rate was 0.71. As the sample size was too small, fewer samples had overall survival rates of 1 and 5 years, so the AUC of 1-year and 5-year were low.

The entire TCGA set included 391 colon adenocarcinoma patient samples. The risk score of each sample was calculated based on Formula (4). The optimal cut-off value of risk score is determined through the surv_cutpoint function of R. The optimal cut-off value of entire TCGA set is 2.02 (Figure 2C). The optimal cut-off value was used to divide the 391 patient samples into two groups, namely, the high- (*n* = 104) and low-risk groups (*n* = 287). Kaplan-Meier survival analysis was used to compare the survival differences between the two groups. The survival analysis results showed differences in survival rate between the two groups ( p<0.001 ; Figure 3E). Overall survival of the high-risk group was lower, and the prognosis was worse. The median survival was more than 10 and 3 years in the low- and high-risk groups, respectively. The three- and 5-year survival rates of the low-risk group were 87% and 78%, respectively. The three- and 5-year survival rates of the high-risk group were 53% and 25%, respectively. Figure 4C shows ROC curves of the entire TCGA dataset. AUC values of the 1-, 3-, and 5-year overall survival rates were 0.76, 0.78, and 0.77, respectively, showing that the prognostic model had good accuracy.

## 4 Discussion

In this study, we found nine prognosis-related immune genes (*ANGPTL4*, *IDO1*, *NOX1*, *CXCL3*, *LTB4R*, *IL1RL2*, *CD72*, *NOS2*, and *NUDT6*), and we calculated the risk score according to their gene expression and correlation coefficient. Previous experiments have shed light on aberration in these immune genes can lead to tumorigenesis and tumour progression.

Nakayama et al. studied the expression of *ANGPTL4* in colorectal cancer and showed that its expression is associated with venous and lymphatic invasion and that it promotes distal metastasis, i.e., *ANGPTL4* is one critical factor of colorectal cancer progression (Nakayama et al., 2011). Huang et al. showed that *ANGPTL4* expression was more frequent in colorectal cancer tissues than in healthy tissues and that it mediates metastasis through the cytoskeleton signalling pathway to promote colorectal cancer invasion and metastasis (Huang et al., 2012).

Bishnupuri et al. found that *IDO1* activity in epithelial cells and kynurenine pathway metabolites activate tumour epithelial PI3K-Akt signalling, which promotes cell proliferation and anti-apoptosis, thus promoting colon tumorigenesis (Bishnupuri et al., 2019). Thaker et al. found that *IDO1* directly promotes tumour growth and tumour epithelial proliferation in a cell-independent manner through the synthesis of uric acid metabolites and activation of β-catenin signalling, showing that *IDO1* can be a potential therapeutic target (Thaker et al., 2013).

Wang et al. found that *NOX1* regulates colorectal cancer cell proliferation and invasion through the *ADAM17-EGFR-PI3K-Akt* axis to promote colorectal cancer metastasis, showing that *NOX1* can also be a potential target in colorectal cancer treatment (Wang et al., 2016). Ohata et al. studied the biological pathways of cancer stem cell proliferation and demonstrated that *NOX1* induces mTORC1 activation through lysosomal S100A9 oxidation and promotes colon cancer proliferation (Ohata et al., 2019).

According to Farquharson et al., insulin and adiponectin can regulate the expression level of *CXCL3* and thereby participate in colorectal cancer tumorigenesis (Farquharson et al., 2012). Liao et al. showed that *CXCL3* can bind to *CXCLR2* on myeloid-derived suppressor cells to promote its migration to the tumour microenvironment (Liao et al., 2019).

*LTB4R* is a receptor of leukotriene B4 and exists in two forms. One is the high-affinity LTB4 receptor *BLT1*, which is expressed in different leukocyte subsets and is responsible for LTB4-dependent leukocyte migration. The other is the low-affinity LTB4 receptor *BLT2*, which is expressed in epidermal keratinocytes and epithelial cells and has wound healing and epidermal barrier functions (Yokomizo et al., 2018). Sharma et al. showed that *BLT1* expression in CD8+T cells plays an important role in tumour metastasis (Sharma et al., 2013). Chheda et al. found that *BLT1* plays a critical role in regulating the migration of cytotoxic T lymphocytes to tumours and anti-tumour immunity (Chheda et al., 2016).

Tomuschat et al. studied the expression of *IL1RL2* in patients with congenital Hirschsprung’s disease (Tomuschat et al., 2017). Their results showed that *IL1RL2* is an important mediator of inflammatory responses and that a significant reduction in its expression can increase inflammatory responses and cause changes in mucosal healing, thereby resulting in susceptibility to Hirschsprung-associated enterocolitis. In addition, Penha et al. showed that *IL1RL2* is expressed in intestinal T lymphocytes and can induce CD4^+^ lymphocyte proliferation, relating to human intestinal diseases (Penha et al., 2016). *CD72* is expressed by various immune, inflammatory and epithelial cells. CD100-CD72 interaction can regulate the intensity of B cell receptor signal pathway, enhance cell activation and maintain immune homeostasis (Wu et al., 2016).

## 5 Conclusion

We downloaded transcriptome data of colorectal cancer healthy tissues from GTEx and then downloaded transcriptome data and clinical data of colorectal adenocarcinoma patients from TCGA. LASSO regression was carried out on DEGs between healthy samples and tumor samples to identify prognosis-related immune genes. Multivariate Cox regression and prognosis-related immune genes (*ANGPTL4*, *IDO1*, *NOX1*, *CXCL3*, *LTB4R*, *IL1RL2*, *CD72*, *NOS2* and *NUDT6*) were used to construct an immune-related prognosis risk score model for colon adenocarcinoma patients. This score was used to divide colon adenocarcinoma patients into high- and low-risk groups. Survival analysis found that the high-risk group had lower overall survival rate and poorer prognosis.

To validate the prognostic value of the model, we computed ROC curves. The model AUC values of the 1-, 3-, and 5-year overall survival rates were 0.76, 0.78, and 0.77, respectively, showing good prediction results for patients’ prognosis. Further multivariate analysis demonstrated that the risk score was an independent prognostic factor. A validation dataset was used to further demonstrate the accuracy of this score. The model also identified immune genes as potential prognostic biomarkers and therapeutic targets in colorectal cancer, however, further validation in clinical trials is required, the mechanism by which immune genes affect cancer progress should be further studied.

## Data Availability

The original contributions presented in the study are included in the article/Supplementary Material, further inquiries can be directed to the corresponding authors.
