# Supplementary material for: Regularized survival learning and cross-database analysis enabled identification of colorectal cancer prognosis-related immune genes
Source: Front Genet. 2023 Feb 23;14:1148470. doi: 10.3389/fgene.2023.1148470 (PMC9995717; doi:10.3389/fgene.2023.1148470)
Supplement: Supplementary file 1 [file Image1.pdf]

Supplementary Material

Regularized Survival Learning and Cross-database Analysis enabled Identification of Colorectal Cancer Prognosis-Related Immune Genes

Dongmei Ai*, Mingmei Wang, Qingchuan Zhang, Longwei Cheng, Yishu Wang, Xiuqin Liu and Li C. Xia

*** Correspondence:** Dongmei Ai: aidongmei@ustb.edu.cn; Li C. Xia: lcxia@scut.edu.cn

1 Supplementary Figures

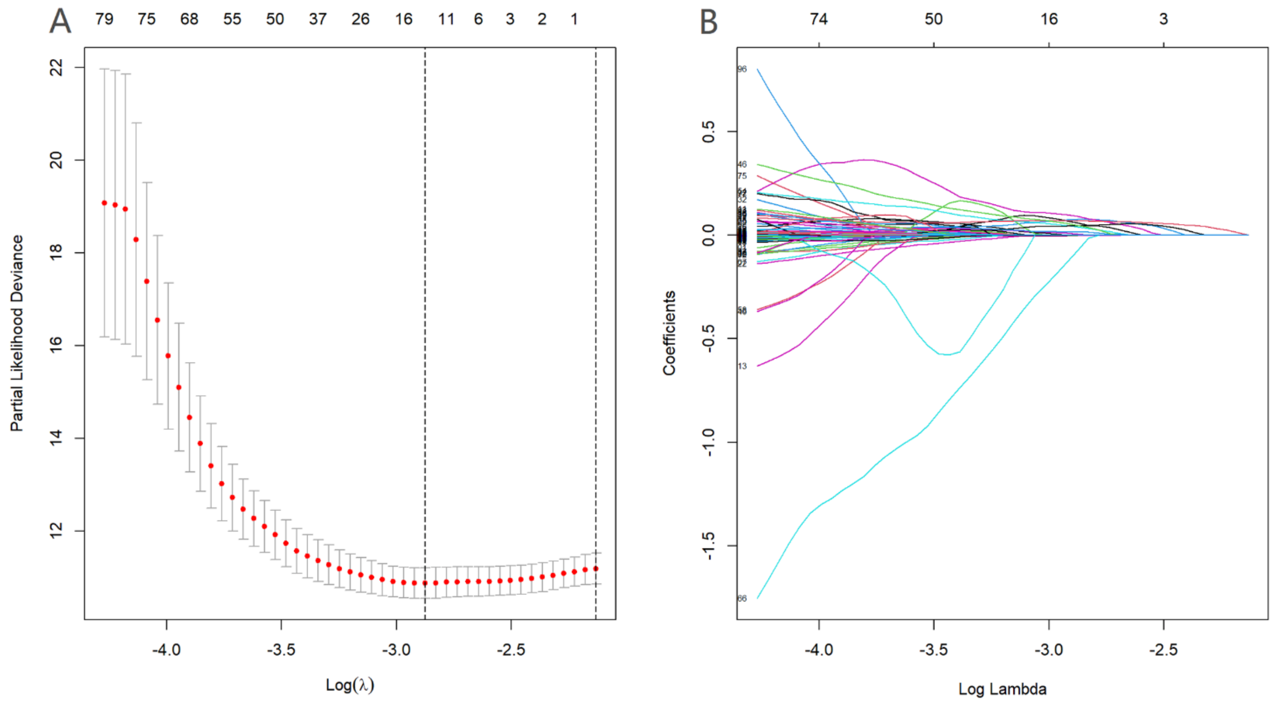

Supplementary Figure S1. The results of screening differentially expressed immune genes by LASSO regression. (A) The x-coordinate is the log lambda. The lambda value is a penalty coefficient, and lambda controls the complexity of LASSO regression. The y-coordinate is the cross-validation error. (B) The x-coordinate is the log lambda value; The y-coordinate is the coefficient. Each line represents a gene. If the coefficient of a gene is 0, then that gene is not included in the subsequent analysis.

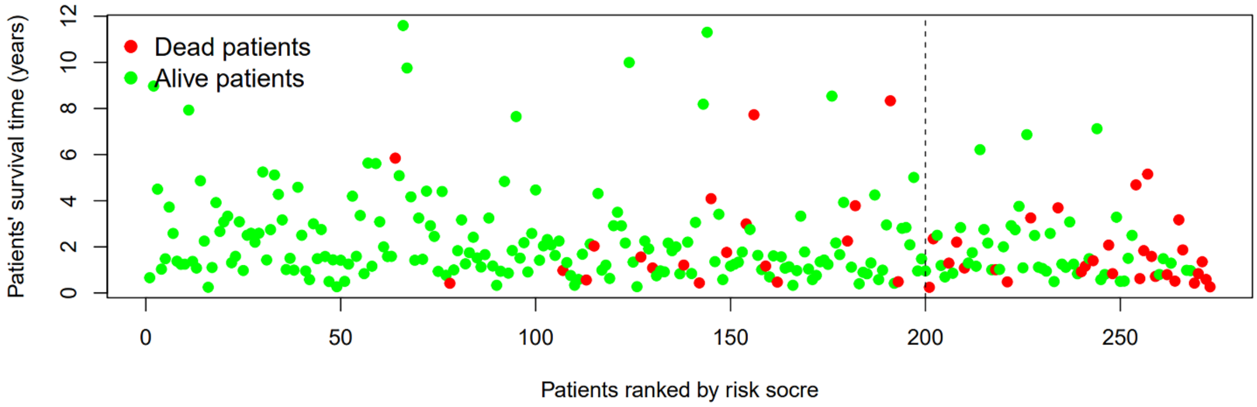

Supplementary Figure S2. Distribution of survival status in the TCGA training dataset. The x-coordinate is the sample, which is ranked by risk score from low to high. The y-coordinate represents the survival period of the sample. Green dots represent alive patients, while red dots represent dead patients.

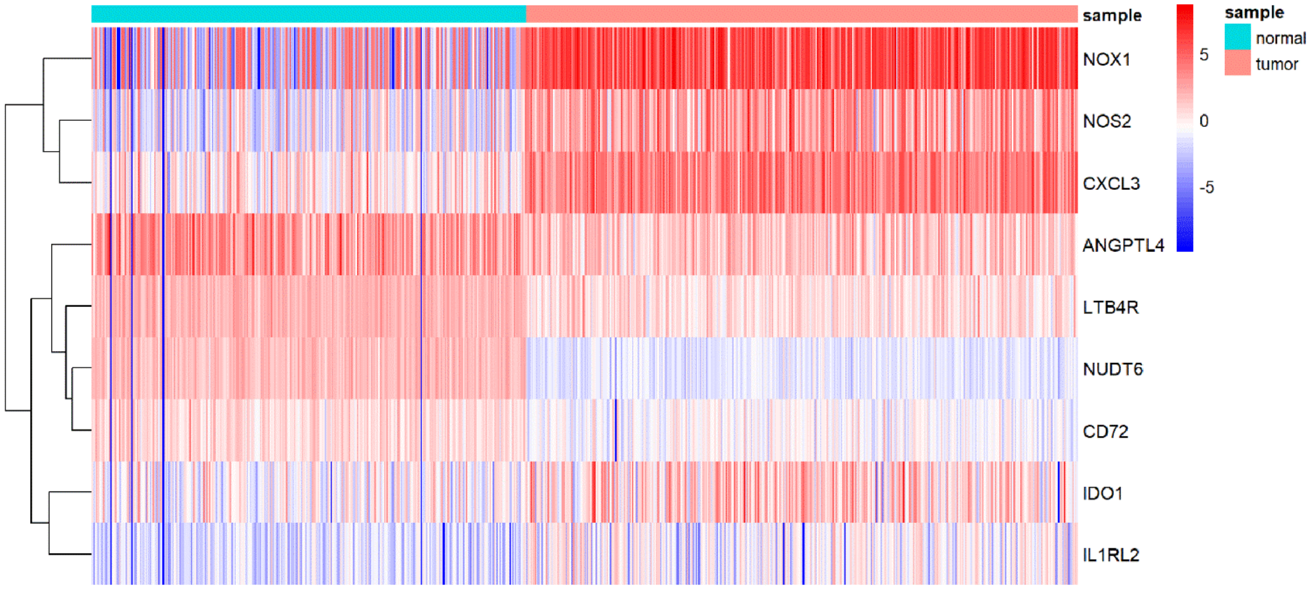

Supplementary Figure S3. Expression heatmap of 9 immune genes in healthy tissues and tumor tissues. The x-coordinate represents the sample, blue represents the healthy group sample, and pink represents the tumor group sample. The y-coordinate represents the gene. In the heatmap, the color changes from blue to red, representing a low gene expression level to a high gene expression level. The gene expression level is $log_2(expression\ value)$.
